# Development status of mouthguard

**DOI:** 10.3389/fdmed.2024.1513223

**Published:** 2024-12-02

**Authors:** Wang Biqi, Wang Xinyu

**Affiliations:** ^1^ Department of Pediadontia, Stomatological Hospital of Jiamusi University, Jiamusi, Heilongjiang, China; ^2^ Department of Oral and Maxillofacial Surgery, Stomatological Hospital of Jiamusi University, Jiamusi, Heilongjiang, China

**Keywords:** mouthguard, characteristic of materials, production method, protection principle, influencing factors

## Abstract

The mouthguard plays a crucial role in preventing damage to the oral and jaw system. However, the popularity of sports mouthguards remains relatively low, and research on sports mouthguards is rather scattered. This paper primarily summarizes the characteristics of materials, production methods, protection principles, and influencing factors of mouthguards, with the aim of providing a theoretical reference for the popularization and application of mouthguards.

## 1 Introduction

The sports mouthguard (MG) is a protective device placed inside the mouth. Primarily worn during participation in sports activities, it can effectively prevent dental and surrounding soft tissue traumas during sports and reduce the risk of maxillofacial fractures, temporomandibular joint traumas, and concussions (1). A significant number of studies have demonstrated (2–4) that the sports mouthguard is the most effective device for reducing or avoiding injuries to the stomatognathic system. However, at present, the popularity of sports mouthguards remains suboptimal, and research on mouthguards is relatively fragmented. This article will summarize the research progress of sports mouthguards in terms of material properties, production methods, protection principles, and influencing factors. It is hoped that while promoting the popularization and application of mouthguards, new prospects and innovative development outlooks will be presented for sports mouthguards.

## 2 Material performance of mouthguards

### 2.1 Material types

Craig & Godwin proposed that mouthguard materials need to possess a certain degree of hardness, impact resistance, stability, tear resistance, water absorption, and appropriate softness and hardness (5, 6). Among these properties, impact resistance mainly depends on the stress absorption capacity and rigidity of the material. Hence, in material selection, performance improvement is primarily targeted at these two characteristics.

Currently applied materials include ethylene vinyl acetate (EVA) copolymer, polyethylene terephthalate glycol (PETG), rubber-like materials, polyurethane, polyvinyl chloride, and acrylic resin, among others.

#### 2.1.1 EVA

EVA (7) (ethylene vinyl acetate) is a polymeric material with outstanding properties such as ease of processing, superb biocompatibility, high flexibility, anti-aging traits, and impact resistance. It is the most commonly employed material for sports mouthguard films (8). Having a small elastic modulus and strong stress absorption capacity, it is inherently elastic. During material deformation, it can prolong the contact time between foreign objects and teeth, thus reducing instantaneous stress. Moreover, it can distribute energy to the periodontal ligament and alveolar bone, minimizing or avoiding damage to the tooth and surrounding soft and hard tissues at the moment of impact. SLIWKANICH et al. (9) found, through a comparison of five commonly used mouthguard materials, that EVA shows the strongest water absorption and impact resistance at 37°C in the oral cavity. Del Rossi et al. (10) believe that the color of EVA film has an influence on material hardness. *in vitro* experiments have indicated that dark films provide a better fit and are more closely conforming to the model.

EVA can also be used to make orthodontic retainers. Their elasticity and memory can stabilize the position of teeth and prevent relapse after orthodontic treatment. They can also be used to fabricate occlusal splints to relieve temporomandibular joint disorders by dispersing the occlusal force and reducing the pressure on the joint. Similarly, they can be applied to produce oral postoperative protective plates to isolate and protect the wound and facilitate healing.

#### 2.1.2 PETG (polyethylene terephthalate glycol)

Polyethylene terephthalate glycol (PETG) (11) is a novel polyester material. It showcases strong toughness, high impact strength, good heat resistance and corrosion resistance, as well as superior environmental protection performance. It is employed in the production of hard films for mouthguards. Moreover, during the processing of this material, when the temperature exceeds the melting point, viscous deformation occurs and it displays strong flow performance, facilitating ease of processing and formation.

PETG are also used to make medical models, such as models of the heart and bones. They can also manufacture prosthetic sockets and serve as the shells of drug-controlled release devices, protecting drug components and allowing the structure to be designed for precise drug release control according to requirements.

#### 2.1.3 FLX series rubber-like materials

Cummins & Spears (12) demonstrated through finite element analysis that low-hardness mouthguards can resist impacts from hard objects (such as steel balls) but are unable to withstand collisions from soft objects (such as boxing gloves). Consequently, the selection of mouthguard materials should possess both the ability to disperse rigid stress and the ability to absorb soft impacts, employing a combination of “hard and soft” measures to safeguard the soft and hard tissues of teeth.

The FLX series rubber-like materials independently developed by China achieve the gradient composite presentation of different softness and hardness properties through 3D digital polymer hybrid printing technology and can be printed in conjunction with rigid materials. Research has indicated (13) that under the same thickness, the FLX mouthguard results in a smaller impact force on teeth. The FLX material can effectively enhance impact resistance while ensuring comfort.

The FLX series of rubber-like materials can also be used to manufacture rehabilitation training aids, such as hand rehabilitation tools. They can also produce spinal orthopedic braces and pressure therapy socks, etc.

#### 2.1.4 Other composite materials

In recent years, numerous scholars in the medical field have made attempts to combine various materials to enhance shock absorption and dispersion effects. This can be specifically classified into: (1) combinations of material types (8, 14); (2) combinations of performance factors other than materials (15); and (3) other alternative materials (16). Westerman et al. (17) discovered through experiments that combining EVA materials of different hardness levels increases its hardness and stiffness. However, in the medical context, it is important to note that the shock absorption rate remains unchanged. This indicates that combining the same material with different hardness values cannot improve the shock absorption effect from a medical perspective.

The Kombiplast film, a material of significance in medical applications, is a thermoformed material with a double-layer structure that is soft on the inside and hard on the outside. Clinically, it can be utilized to fabricate dental splints, soft and hard occlusal pads, sports mouthguards, teeth whitening trays, and orthodontic auxiliary instruments. From a medical standpoint, the main component of its inner layer is EVA, while the main component of the outer hard film is PETG. Some medical researchers have found (18) that the elastic modulus of the inner layer of the Kombiplast film is significantly lower than that of self-curing resin material of the same thickness. Moreover, the hardness of the outer layer of the film is greater than that of self-curing resin, which has important implications for medical applications. It can be also used to fabricate dental appliances for treating sleep apnea syndrome to improve breathing by adjusting the mandible position. They can also produce correctors for children's oral bad habits to guide normal oral development. Additionally, they can be applied to make positioning aids for oral and maxillofacial radiotherapy, etc.

Motoyoshi M et al. (19), in the realm of medical research, developed a two-component, five-layer sheet material (referred to as ND). This material is composed of a surface layer of PO5% and EVA 40%; and a middle layer of PO10%, EVA 40%, and PO 5%. From a medical evaluation perspective, the results of impact tests and shock absorption tests show that ND has excellent shock absorption performance and dispersion ability, which can be beneficial for protecting oral and dental structures.

Hiroshi Churei et al. (20) evaluated the application of glass fiber reinforcement methods in mouthguard materials from a medical engineering perspective. It was found that the bonding strength of EVA-based glass fiber reinforced materials is significantly improved and the bending performance is enhanced, which can enhance the durability and effectiveness of mouthguards in medical settings.

Chao Huang et al. (21) introduced non-Newtonian materials into mouthguard materials, which is a significant innovation in medical materials. The prepared mouthguard with shear strengthening properties (SSM) shows that it has outstanding shock absorption ability and a soft feel, providing better comfort and protection for patients.

Jing Zhou et al. (22) developed a remodelable shear-strengthened mouthguard (RSSM), which has a shear-strengthening effect and excellent shock absorption ability. From a medical application standpoint, it can absorb more than 90% of the energy. Even if its thickness is reduced by half, compared with commercial mouthguard materials (Erkoflex and Erkoloc-pro), it can reduce the transmitted force by approximately 25% and extend the buffering time by about 1.6 times. At the same time, it exhibits good plasticity, stability, and biocompatibility, making it a promising material for medical use.

### 2.2 Material structure

In addition to developing various composite materials, a large number of experiments have been conducted to improve the structural design of mouthguards to enhance their impact resistance.

Sarac et al. (23) used triangular laser sensors to measure the shock absorption effect of EVA mouthguards of different thicknesses and with labial filling materials (PETG, nylon mesh, air filling). The experiment proved that increasing the material thickness and adding lip filling materials can improve the shock absorption ability when small hard objects collide. Pinho AC et al. (24) evaluated the mechanical properties of three sandwich structures using different polymer materials (ABS-TPU-ABS, PMMA-TPU-PMMA, HIPS-TPU-HIPS). Among them, ABS-TPU-ABS has the highest resilience value among all material combinations.

Joao Paulo Mendes Tribst et al. (25) conducted a biomechanical analysis of customized mouthguards reinforced with laminates of different elastic moduli during maxillofacial trauma simulation. It was found that using reinforcement inside the customized mouthguard can change the stress generated on the buccal surface of enamel, but it will not improve the root, periodontal ligament or bone tissue. Andrew Shelley et al. (26) conducted a systematic evaluation of the effectiveness of hard inserts in sports mouthguards. The statistical results are contradictory, and the efficacy of hard inserts in sports mouthguards has not been proven.

An impact-resistant multifunctional mouthguard (27) has added a cushion on the lower surface of the alveolar ridge, increasing the contact friction coefficient between the mouthguard and teeth, which is beneficial for the occlusion of mandibular teeth and the stability of the mouthguard.

## 3 Types and production methods of sports mouthguards

### 3.1 Types of mouthguards

Sports mouthguards are generally divided into three major categories: ready-made mouthguards, intraoral molded mouthguards, and personalized custom mouthguards. The World Dental Federation recommends using well-fitting mouthguards, and the best choice is personalized custom mouthguards (28). It has many advantages, including a high degree of protection, convenient storage, good fit and comfort, and it does not affect the user's breathing function (29).

### 3.2 Production methods of mouthguards

#### 3.2.1 Direct molding method

Latex, rayon or nylon fiber-reinforced latex is directly formed on a plaster model.

#### 3.2.2 Lost wax method

Plasticized acrylic resin mixed with powder and liquid is used to make it by the lost wax method, just like making a complete denture base.

#### 3.2.3 Thermoforming method

There are various types of equipment for making customized mouthguards. Among them, pressure forming machines and vacuum forming machines are two relatively common types (30). A pressure forming machine forms materials by applying a certain pressure. It can provide a relatively stable forming pressure and may be more suitable for the production of mouthguards made of materials that require higher pressure for forming or have high shape precision requirements. On the other hand, a vacuum forming machine forms materials by using the principle of vacuum suction, adsorbing the heated material onto the mold. This method is relatively gentle and has a good forming effect on some soft and easily deformable materials. Moreover, it can better maintain the original properties of the materials during the forming process.

Pressure forming machines and vacuum forming machines have differences in working principles and forming effects (31). In terms of principle, a pressure forming machine acts on the material by external pressure, while a vacuum forming machine makes the material fit the mold by internal vacuum negative pressure. In terms of forming effects, a pressure forming machine can make the material fill the details of the mold more closely, which is suitable for making mouthguards with complex structures and high precision requirements; a vacuum forming machine can produce mouthguards with relatively smooth surfaces and less internal stress in the material, which has advantages for making mouthguards with high requirements for surface quality and material properties.

It cannot be simply said that the mouthguards produced by a pressure forming machine or a vacuum forming machine are of higher quality. This depends on various factors, such as the design requirements of the mouthguards and the characteristics of the materials used. If emphasis is placed on the structural precision and complex shape forming of the mouthguards, a pressure forming machine may be more appropriate; if surface quality, maintenance of material properties, and forming effects on soft materials are emphasized, a vacuum forming machine may have more advantages. In actual production, sometimes the two forming methods are combined according to specific circumstances to achieve the best forming effect and mouthguard quality.

Yamada & Maeda (32) pointed out that the most suitable temperature range for EVA molding is 80–120°C, and the molding process should be completed before reaching the lower limit of the temperature. Geary & Kinirons (33) tested the thickness change of the EVA film after compression molding under a series of conditions. They believed that the controllable conditions that can affect the quality stability of customized mouthguards include: model height, inclination, shape, model temperature, model position on the compression molding plate, plasticizing time and suction method.

Lamination technology can be applied in the production of mouthguards. Kenyon & Loos (34) found that double-layer laminated mouthguards can design patterns on the mouthguard, have a variety of color choices, and the thickness can be controlled. Miura et al. (35) found that double-layer laminated mouthguards have less stress accumulation in long-term deformation resistance.

#### 3.2.4 3D printing method

Li et al. (36) used 3D printing technology to make mouthguards. By comparing with traditional mouthguards, it was found that traditional mouthguards may cause the second molar to have a single occlusal contact, while 3D printed mouthguards can evenly distribute the occlusal force, improve comfort and avoid stress concentration.

Unkovskiy et al. (37) developed a 3D printed double-layer custom sports mouthguard. After intraoral scanning and digital design, two computer-aided manufacturing technologies, Polyjet 3D printing and silicone resin dripping, are used. The final product has a harder material on the outer layer to enhance its protective function, and the soft material on the inner layer can better fit the mucosa and teeth.

Arfi Yohan et al. (38) compared the shock absorption capabilities of 3D printed custom mouthguards, industrial mouthguards and thermoformed EVA mouthguards through *in vitro* research experiments. The research shows that 3D printed mouthguards show better shock absorption capabilities, are least affected by repeated mechanical tests, and have the smallest thickness change.

Maciej Trzaskowski et al. (39) evaluated the mechanical properties of four flexible polymer 3D printing materials. The experiment shows that EnvisionTEC's Keyortho IBT material is most suitable for making mouthguards. Nasrollahzadeh (40) combined multiple methods such as finite element simulation, additive manufacturing and impact testing for mouthguard research. The results show that the 3D printed mouthguard combined with the spacer guide rail made of Key IBT resin and the insert made of ST1400 resin can enhance the tooth protection ability and significantly improve various properties.

Tamaki Hada (41) proposed 4D printing technology, which adds the concept of time on the basis of 3D printing technology, that is, creating a molded object that restores its predefined shape as it responds to external stimuli. In the experiment, a double-layer system was used for the material: the inner layer was made of TPU with a high elastic modulus to protect the dentition; the outer layer was composed of a composite of TPU and SMP. SMP is a shape memory polymer with shape memory effect (SME). This technology simplifies the production process and solves the problem of reduced fit of the mouthguard due to deformation.

## 4 Protection principle of sports mouthguards

Chapman (42) proposed that the protective effect of sports mouthguards is mainly achieved through three types of buffering and conduction: Type I protection is the buffering and absorption of impact force; Type II protection is the dispersion of maxillary impact force; Type III protection is the dispersion of intermaxillary impact force.

The thickness of the mouthguard only involves Type I and Type II protection. Type III protection is achieved through the contact between the mandibular dentition and the mouthguard. Some studies have found (43) that when the maxillary central incisor is impacted from the front, Type I and Type III protection are significant; when the maxillary molar is impacted laterally, Type I and Type II protection are significant; when the chin is impacted, Type II and Type III protection are significant.

## 5 Influencing factors of sports mouthguards

### 5.1 Thickness

The thickness of sports mouthguards is very important for protective performance and can directly affect the impact absorption capacity.

Westerman et al. (8) believe that the optimal thickness of EVA material is about 4 mm. If the thickness increases, although the stress absorption capacity is slightly increased, for the wearer, the comfort and acceptability will decrease. Maeda et al. (44) believe that the minimum thickness of EVA material to absorb sufficient energy is 4 mm. Yamada et al.'s (45) research also proves that at least a thickness of 3 mm is needed to significantly reduce the deformation under impact force. Compared to the position of the palatal edge of the anterior teeth, thickness is more important for reducing horizontal impact force. TAKAHASHI M et al. (46) conducted research on film pretreatment and found that compared with vacuum suction after peripheral clamping of the film, vacuum suction after four-point clamping of the film reduces the thickness reduction ratio of the anterior teeth area. Making a V-shaped groove on the original film in advance also helps reduce the thickness change ratio of the anterior teeth area.

### 5.2 Occlusion

Occlusal contact is the result of the synergistic action of teeth, mandible, nerves and muscles, and is an important manifestation of the interconnection of various parts of the stomatognathic system. Balanced occlusion can not only improve the protective effect but also increase comfort (47, 48). Takeda et al. (49) compared the influence of lower anterior teeth occlusal contact on the protective effect of sports mouthguards through pendulum impact tests. The results showed that when the lower anterior teeth are fully occlusally contacted with the sports mouthguard, the supporting force of the mandible is transmitted to the maxilla through the sports mouthguard, significantly improving the protective effect of the sports mouthguard. The strong occlusal force combines the upper and lower jaws into a firm whole. When the maxillofacial region is impacted, the impact force is transmitted to the opposite jaw through occlusal contact, reducing the local effect of the impact force and reducing trauma.

### 5.3 Shape design

When designing the shape of the mouthguard, it should not affect the breathing and speech functions as much as possible to improve the comfort of wearing for patients. McClelland et al. (48) proved that when the labial side of the mouthguard extends to 2 mm from the vestibular transition, the occlusal contact is balanced, the buccal margin is rounded, and the palatal margin is transitional, the wearing comfort of the mouthguard is significantly increased. Maeda et al. (50) studied the influence of appearance design and edge treatment on the wear resistance and deformation resistance of mouthguards *in vivo*. It is pointed out that by trimming the palatal edge to the cervical margin, smoothing all edges and adjusting the occlusion, the comfort of the mouthguard, the degree of affecting breathing and the degree of affecting swallowing are all improved.

### 5.4 Personal protective equipment legislation

In terms of personal protective equipment regulations, as a specific oral protective article, mouthguards need to comply with relevant safety and quality standards. For example, in some regions, the materials of mouthguards are required to meet biocompatibility standards to ensure the safety of use in the oral environment and avoid allergic or other adverse reactions in users. At the same time, there are also certain regulatory requirements for the protective performance of mouthguards, such as impact resistance test standards, to ensure that they can effectively prevent oral and maxillofacial injuries during sports. Different countries and regions may regulate the production, sales, and use of mouthguards according to their own regulatory systems. Manufacturers need to ensure that their products meet the corresponding regulatory requirements to be legally circulated in the market.

## 6 Conclusion

Sports mouthguards can effectively prevent and reduce potential maxillofacial traumas in various sports. As dentists, they should have a comprehensive understanding of all aspects of sports mouthguards, such as various production materials, manufacturing methods and molding technologies, protection principles and influencing factors. Additive manufacturing (AM) and 3D printing technology can be used to make mouthguards. Compared with traditional custom mouthguards, using such technologies may produce sports mouthguards with higher fitting tightness and impact resistance. In the future, more research on innovative materials and structural design is needed to provide more theoretical references for the clinical application of mouthguards.
